# Enhancing Photocurrent Performance Based on Photoanode Thickness and Surface Plasmon Resonance Using Ag-TiO_2_ Nanocomposites in Dye-Sensitized Solar Cells

**DOI:** 10.3390/ma12132111

**Published:** 2019-06-30

**Authors:** Muhammad Quisar Lokman, Suhaidi Shafie, Suraya Shaban, Fauzan Ahmad, Haslina Jaafar, Rizuan Mohd Rosnan, Hafizal Yahaya, Shahrum Shah Abdullah

**Affiliations:** 1 Malaysia-Japan International Institute of Technology (MJIIT), Universiti Teknologi Malaysia, Kuala Lumpur 54100, Malaysia; 2 Institute of Advanced Technology, Universiti Putra Malaysia, UPM Serdang, Selangor Darul Ehsan 43400, Malaysia; 3 JEOL (Malaysia) Sdn. Bhd, Petaling Jaya, Selangor Darul Ehsan 47301, Malaysia

**Keywords:** dye-sensitized solar cells, photoanode thickness, silver nanoparticles, surface plasmon resonance, Ag-TiO_2_

## Abstract

This study investigated the different thicknesses of TiO_2_ photoanode films and the effect of surface plasmon resonance (SPR) of Ag-TiO_2_ nanocomposites on the current-voltage (I–V) performance of dye-sensitized solar cells (DSSC). The TiO_2_ layer was deposited using the doctor blade technique and the thickness of the TiO_2_ films was controlled by using a different number of Scotch tape layers. The silver nanoparticles (AgNP) were synthesised using a chemical reduction method and the concentration of sodium citrate as a reducing agent was varied from 4 to 12 mM to study the effect of citrate ion on the size of the nanoparticles. Ag-TiO_2_ nanopowder was prepared by adding pure anatase TiO_2_ powder into AgNP colloidal solution. The mixture was left to dry for 24 h to obtain Ag-TiO_2_ powder for paste preparation. The three-layer Scotch tape, with thickness of 14.38 µm, achieved a high efficiency of 4.14%. This results showed that three layers was the optimal thickness to improve dye loading and to reduce the charge recombination rate. As for the Ag-TiO_2_ nanocomposites, 10 mM of AgNP, with a mean diameter of 65.23 nm and high efficiency of 6.92%, proved that SPR can enhance the absorption capability of dye and improve the photon-to-electron generation.

## 1. Introduction

Dye-sensitized solar cells (DSSC) have been an object of research since they were discovered in 1991 by Grätzel and O’Regan as an alternative to conventional solid-state photovoltaic (PV) cells. DSSC have several unique advantages, such as low-cost production, being environmentally friendly, flexible, lightweight, and showing good performance under lowlight illumination conditions [1,2]. In the conventional solid state PV, also known as first-generation PV cells, photon absorption and electron transport would occur in the crystalline silicon p-n junctions [3]. This solid state cell is a frontier in PV technology that has contributed to 80% of the world PV market. However, the first-generation PV cells still face the issue of high production cost and complex fabrication process [4]. To overcome the issue of high production cost, thin-film solar cells based on amorphous silicon and cadmium telluride (CdTe) materials were introduced. Highly flexible thin-film solar cells are categorised as second-generation PV cells that have drastically reduced the cost of production. However, the thin films have shown low performance due to the low quantum efficiency of amorphous silicon and the high toxicity of CdTe, which require high precaution during manufacturing [4]. It is predicted that DSSC will be among the next generations of PV that lies in third-generation PV cells. In DSSC, two separate processes occur in different mediums. A ruthenium complex dye is adsorbed onto TiO_2_ nanoparticles to be used as a light absorption material. The excited electron will be injected into the nanoporous TiO_2_ network and regenerated by tri-iodide electrolyte to complete the cycle of electron regeneration [5]. The DSSC cells are able to achieve a power conversion efficiency of 13% using D-π-A porphyrin dye sensitizer and cobalt-based redox mediator [6]. However, their efficiency is still lower compared to other generation PV cells [7]. Thus, numerous studies are being conducted to find a solution to overcome the issue of low efficiency.

Sengupta et al. reviewed several parameters in improving the photoanode to obtain high power conversion efficiency in DSSC [8]. In their review, the thickness of the photoanode film and the metal-TiO_2_ nanocomposites were found to be major contributors to the improvement of efficiency. In DSSC, the thickness of TiO_2_ films plays an important role in the high generation of charge collection and reduction of the charge recombination rate [9]. Kumari et al. studied the photovoltaic properties of DSSC at different photoanode thicknesses [10]. They reported that ~12 µm thickness was enough to enhance the photo-generation activity in DSSC, as well as to reduce the charge recombination. Ito et al. also reported the effect of photoanode thickness on the efficiency of DSSC using screen printing techniques. In their report, the best thickness ranged from 12 to 20 µm to gain higher efficiency and photocurrent generation [11]. Metal nanoparticles are also one of the factors that can improve the photocurrent and power-conversion efficiency. Metal nanoparticles, especially silver (Ag) and gold (Au), are able to enhance light absorption and broaden the light spectrum of the dye through surface plasmon resonance (SPR). This metal nanoparticle can function as a light-scattering mechanism that can increase the number of optical pathways, allowing light to stay longer and increase light absorption [6]. Compared to Au, Ag nanoparticles (AgNP) have a high scattering efficiency and energy band. Therefore, AgNP is one of the best candidates to enhance power-conversion efficiency by utilising the SPR effect. Photipitak et al. (2011) studied the effect of different AgNP sizes on the efficiency enhancement of DSSC. The Ag-TiO_2_ nanocomposites were prepared by immersing TiO_2_ film into AgNO_3_ solution and irradiated under ultraviolet (UV) light. The UV light irradiation exposure time was varied from 5 to 240 min to study the variations of AgNP size. In their study, the optimal efficiency that can be achieved was 4.76% with 19.16 nm AgNP [12].

In this work, the I–V characteristic performance was studied by varying the thickness of TiO_2_ films and the size of AgNP to improve the power-conversion efficiency. TiO_2_ was deposited using the doctor blade method and the thicknesses of the TiO_2_ films were controlled using different numbers of Scotch tape layer. Ag-TiO_2_ nanoparticles were prepared by adding pure anatase TiO_2_ powder into colloidal AgNP. The AgNP particle sizes were varied by varying the concentration of sodium citrate as a reducing agent. Finally, the solar cells were tested under AM 1.5 solar simulator to measure the I–V characteristic.

## 2. Materials and Methods

### 2.1. Chemicals

All chemicals were used without any further purification. Silver nitrate (AgNO_3_) was purchased from Systerm. Tri-sodium citrate powder was purchased from R&M Chemicals (Selangor, Malaysia), while the rest of the chemicals used in this study were purchased from Sigma Aldrich.

### 2.2. Synthesis of Silver Nanoparticles (AgNP)

The colloidal AgNP was prepared using the chemical reduction technique. First, 0.05 mmol of silver nitrate was dissolved in 50 mL of filtered de-ionized (DI) water, followed by vigorous stirring on a hot plate until the solution came to a boil. Then, 5 mL of aqueous tri-sodium citrate was added drop by drop at a rate of 1 drop/s, with constant stirring at 500 rpm for 15 min until the solution changed from colourless to dark yellow. Next, the heating element was turned off, while the solution was being stirred continuously until it reached ambient temperature. The AgNP colloid was kept in a refrigerator and wrapped with aluminium foil to prevent direct exposure to sunlight. To study the effect of particle sizes, the concentration of tri-sodium citrate as the reducing agent was varied at 6, 8, 10, and 12 mM.

### 2.3. Preparation of TiO_2_ and Ag/TiO_2_ Photoanode

**Figure 1:**
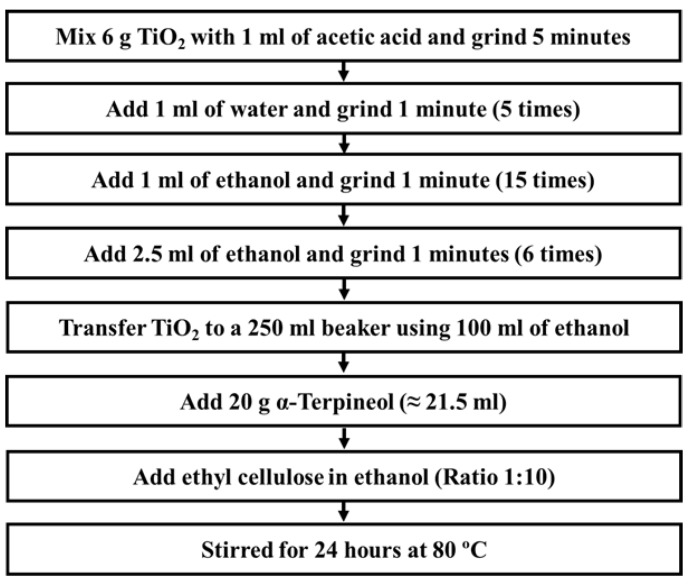
Preparation of TiO_2_ paste.

TiO_2_ paste was prepared as shown in Figure 1. Prior to the preparation, anatase TiO_2_ powder was sintered at 450 °C for 30 min to remove any moisture and inorganic impurities. At each step, liquids were added at a rate of 1 drop/s into a porcelain mortar. The preparation was done in ambient condition at room temperature. The TiO_2_ was dispersed in 100 mL ethanol and transferred into a 250 mL beaker, followed by stirring at 800 rpm. Then, the TiO_2_ dispersion was ultrasonicated for 30 min for better dispersion of TiO_2_ into the paste. Lastly, α-terpineol and ethyl cellulose in ethanol were added, followed by stirring for 24 h at 80 °C. The TiO_2_ paste was slowly evaporated until it reached a suitable viscosity for the deposition process. While preparing the paste, only plastic spatulas and glass slides were used to avoid any surface recombination by iron ions and iron oxide that usually exists in steel spatula.

Ag-TiO_2_ paste was prepared in a similar manner as the pure TiO_2_ paste. Prior to this, 6 g of TiO_2_ powder was added into 60 mL of AgNP colloid, which was then heated at 100 °C for 10 min. The resulting powder was filtered using filter paper and dried in an oven at 80 °C for 12 h. The photoanode was prepared by depositing the TiO_2_ paste on clean fluorine-doped tin oxide (FTO) glass substrates (2.5 × 2 cm^2^) using the doctor blade method and the thickness of the photoanode was controlled by increasing the number of Scotch tape layers. In this experiment, adhesive Scotch tape (3M, Scotch Magic Tape) with thickness of approximately 52 µm was placed on the edges of the conductive sides of the FTO glass to create a 1 × 1 cm^2^ rectangular area for TiO_2_ paste deposition. A small amount of TiO_2_ paste was then applied on the masked top edge of the FTO glass and spread across the unmasked area using a microscope slide. Then, the Scotch tape was removed, leaving an uncoated area of the FTO glass, which was used as the electrical contact for solar measurement. The TiO_2_-coated film was kept in a clean box for 3 min to ensure that the paste can relax to reduce surface irregularity. Then, it was dried for 6 min on a hotplate at 125 °C to evaporate ethanol. Finally, the photoanode was sintered at 450 °C for 30 min and was left to cool down to 80 °C. 

### 2.4. Solar Cells Fabrication

**Figure 2:**
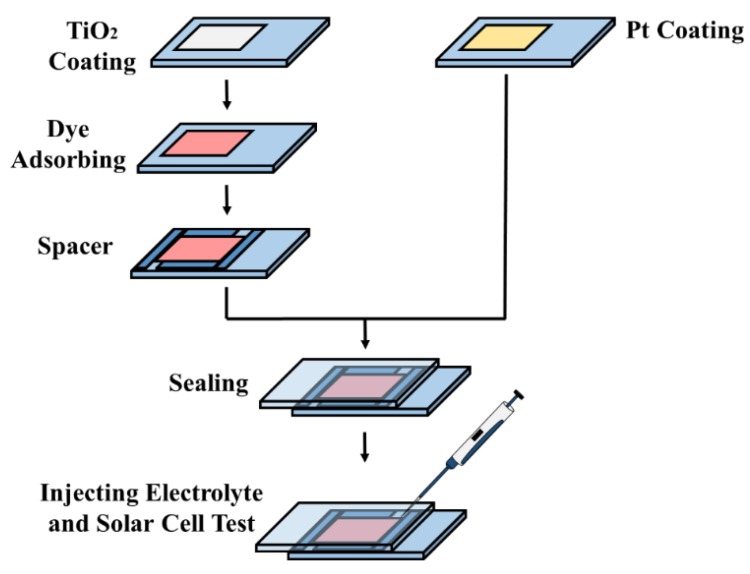
Dye-sensitized solar cells (DSSC) assembly.

Upon reaching 80 °C after the cooling period, the photoanode was immersed in 0.2 mM of N719 dye solution in a mixture of an equivalent ratio of tert-butyl alcohol and acetonitrile at ambient temperature for 20 h for sensitizing. To prepare the counter electrode, 2 mM of H_2_PtCl_6_ in isopropanol was spin-coated on FTO glass and treated at 450 °C for 30 min. The dye-covered photoanode and Pt counter electrode were assembled into a sandwich structure and sealed with 60 µm Surlyn polymer spacer. An electrolyte composed of 0.05 M iodine, 0.1 M lithium iodide, 0.5 M 4-tert-butylpyridine, and 0.6 M ethyl-methyl-imidazolium iodine in acetonitrile was mixed together using a magnetic stirrer. A drop of electrolyte was injected into a solar cell and immediately tested under a solar simulator. Figure 2 illustrates the schematic diagram of DSSC assembly. 

### 2.5. Characterisation and Measurement

The nanoparticle morphologies of AgNP and Ag-TiO_2_ were observed using a transmission electron microscopy (TEM) (JEOL, JEM-2100F, Tokyo, Japan). Energy-dispersive X-ray spectroscopy (EDS) was done using TEM. The particle size and size distribution were measured using a Zetasizer Nano (Malvern Panalytical, ZSP 5600, Royston, UK). Absorption spectrum was analysed using an ultraviolet-visible-near infrared (UV–Vis-NIR) spectrophotometer (Pelkin Elmer, Lambda 750). An X-ray diffraction (XRD) diffractometer (Malvern Panalytical, Empyrean, Royston, UK) was used to study the crystanallity of AgNP, TiO_2_, and Ag-TiO_2_. The I–V characteristic of the DSSC was evaluated using a Keithley 2450 SourceMeter, and a solar simulator (Grating. Inc., Albuquerque, NM, USA) equipped with a Xenon lamp and an air mass 1.5 global filter. The solar simulator was calibrated to an intensity of 100 mW/cm^2^ (1 Sun) using a light meter.

## 3. Results and Discussions

### 3.1. Charaterisation of AgNP and Ag/TiO_2_

**Figure 3:**
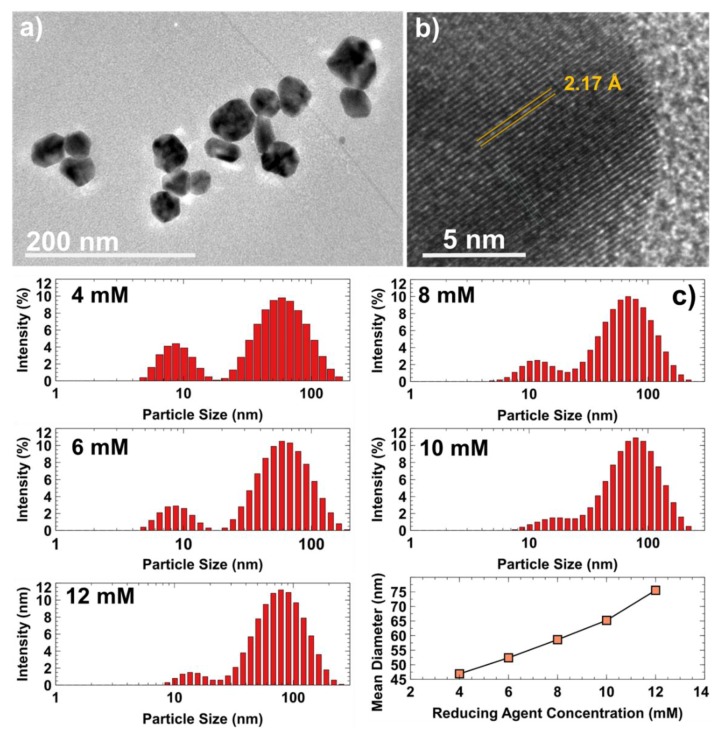
(**a**) Transmission electron microscope (TEM) and (**b**) high-resolution transmission electron microscope (HR-TEM) image of AgNP colloidal solution and (**c**) particles size distribution for different sodium citrate molarity. The mean diameter for 4 mM = 46.89 nm, 6 mM = 52.39 nm, 8 mM = 58.61 nm, 10 mM = 65.23 nm and 12 mM = 75.54 nm.

To observe the morphological structure of the synthesised AgNP, a TEM is used and the image is shown in Figure 3a. The TEM images show that most of the AgNP were in a quasi-spherical geometry and mono-dispersed. The crystalline nature of a single particle was further studied by measuring the lattice fringes to find the d-spacing using a high-resolution transmission electron microscope (HR-TEM). The lattice spacing for the synthesised Ag is shown to be 2.17 Å, which is comparable with a previous report [13]. Figure 3c shows the particle size distribution for different sodium citrate (reducing agent) concentrations, which were determined using a particle size analyser. Figure 3c shows that the synthesised particles size is in the range of 5 to 100 nm. The mean diameter was observed to be increased from 46.89 to 75.54 nm when the reducing agent was increased from 4 mM to 12 mM, respectively. As we can see in the figure, most of the particles were in a range greater than 40 nm, due to slow rate in the citrate reduction method. Therefore, it is difficult to determine the changes of particles sizing towards the different concentration of reducing agent. Thus, mean diameter (Δ nm) was used to observe the changes [14]. A diameter that is larger than 70 nm may contribute to the agglomeration of particles due to low sufficient force to prevent the agglomeration [15]. The high molarity of the reducing agent could create strong attractions between atoms, where the Ag atoms would attract each other and lead to the agglomeration of Ag particles.

**Figure 4:**
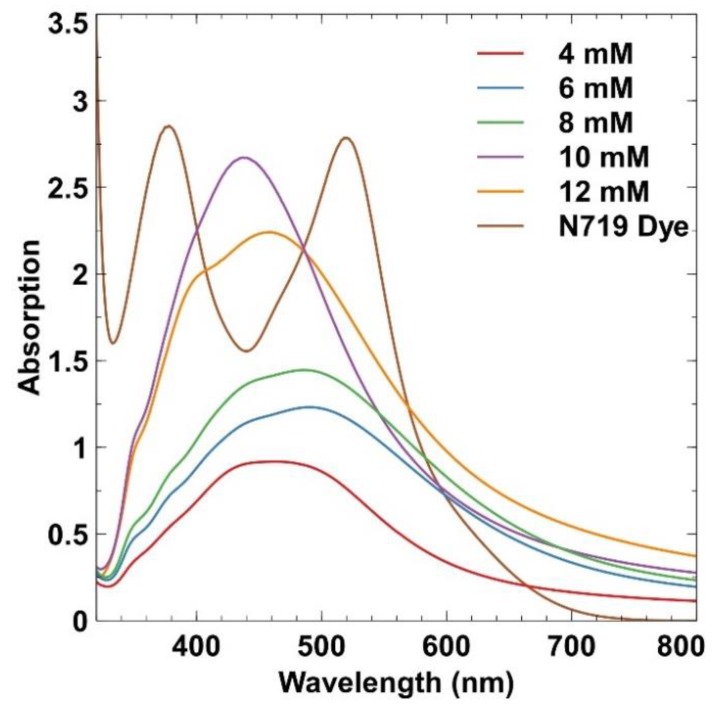
Ultraviolet–visible (UV–Vis) absorption spectrum for N719 dye and different concentration of sodium citrate measured using UV-Vis spectrophotometer.

Figure 4 shows the optical absorption of N719 dye and the effect of AgNP sizes at different sodium citrate concentrations using a UV-Vis spectrophotometer. The synthesised AgNP samples were dispersed into filtered DI-water with a ratio of 1:10 (mg:mL) before being filled into the quartz cuvette. The N719 dye was added, with a similar ratio as AgNP, into an equivalent ratio of acetonitrile and tert-butyl alcohol as a solvent. The optical absorption of N719 dye occurred in two wavelengths at 382 and 523 nm. However, in between the two peaks low absorption of light would occur, especially at the 445 nm region, which can reduce the photon to electron generation. To occupy this region, AgNP was introduced to optimise light absorption. In the UV-Vis spectrum, the synthesised AgNP exhibited a strong absorption within the range of 420 to 500 nm, which is the characteristic spectrum of SPR for Ag [15]. A broad bandwidth of plasmonic absorption is observed in Figure 4, and SPR peak shifted from 463 to 494 nm for 4 to 8 mM, respectively, which indicates red-shifting. This shift in SPR peak was due to the increasing particle size. When the molarity of the reducing agent was increased, the particle size had also increased, which could promote more nucleation. As the concentration of the reducing agent was further increased to 10 mM, the SPR peak started to undergo a blueshift at 442 nm and a narrowed bandwidth was observed. At 10 and 12 mM of sodium citrate concentrations, the SPR peaks of both concentrations began to occupy the lower absorption peak of N719 dye at 445 nm. This result showed that light absorption spectrum can be enhanced, with a high potential of increasing the photocurrent generation. The increased SPR absorption with the increasing reducing agent concentration indicated the occurrence of a greater amount of Ag⁺ reduction [16].

**Figure 5:**
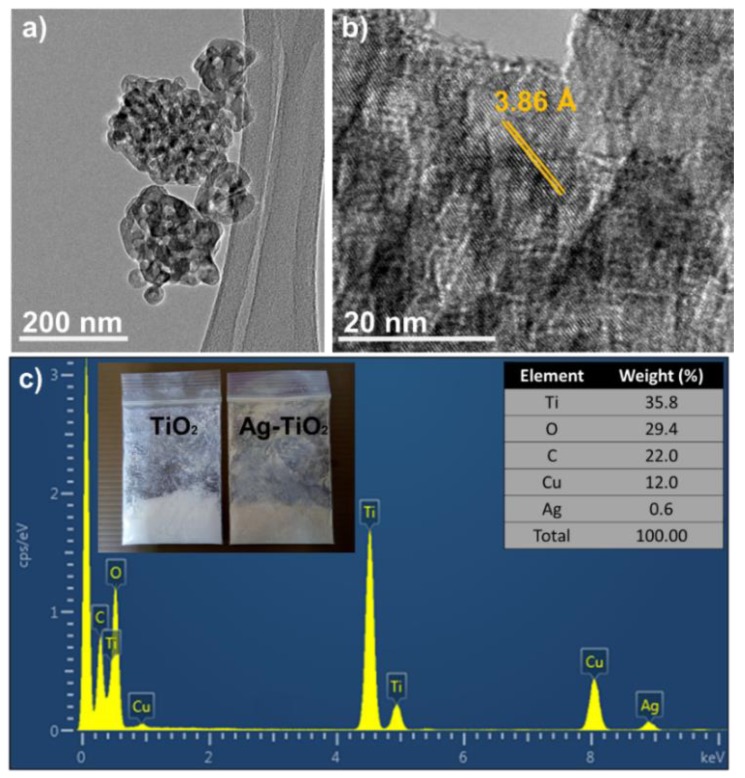
(**a**) TEM, (**b**) HR-TEM image of Ag-TiO_2_ and (**c**) energy dispersive X-ray spectroscopy (EDS) spectrum for determination of elemental composition Ag-TiO_2_ nanocomposites.

Figure 5a,b show the TEM and HR-TEM images of Ag-TiO_2_ nanoparticles dispersed in an ethanol solution with 10 mM of reducing agent. The dark spots with quasi-spherical shapes in the TEM images indicate the presence of AgNP on the TiO_2_ nanoparticles. The HR-TEM image demonstrates the lattice spacing for Ag-TiO_2_ nanoparticles with spacing of approximately 3.86 Å. To further confirm the presence of AgNP on the TiO_2_ nanoparticles, EDS was performed to investigate the elemental composition of Ag-TiO_2_. Figure 5c shows the EDS spectrum of Ag-TiO_2_ using the back-scatted electron method and the inset depicts the physical image of pure TiO_2_ and Ag-TiO_2_ nanoparticles. In the EDS spectrum, the high peak corresponds to titanium (Ti) element, with a peak percentage weight of 35.8%, followed by oxygen (O) with 29.4%, which indicates that TiO_2_ nanoparticles are present. Peaks of carbon (C) and copper (Cu) represent the use of copper grid and lacey carbon. Lastly, the low weight percentage of silver (Ag) at 0.6% can prove that Ag are present in the Ag-TiO_2_ nanocomposites.

**Figure 6:**
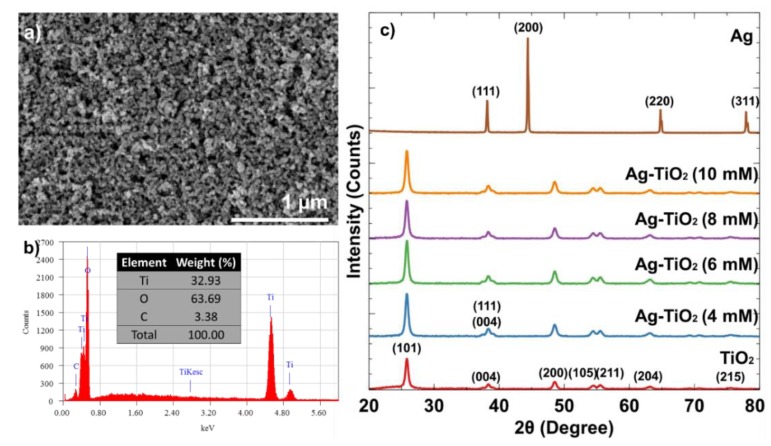
(**a**) FESEM image, (**b**) EDS spectrum of TiO_2_ film and (**c**) X-ray diffraction (XRD) pattern for AgNP, anatase TiO_2_ and Ag-TiO_2_ nanopowder at different of AgNP sizes.

The image surface morphology of pure TiO_2_ film is captured using the field-emission scanning electron microscope (FE-SEM), as displayed in Figure 6a. The 40 kX magnification image, with an accelerating voltage of 2 kV, shows that the TiO_2_ particles were spherical in shape, with highly nanoporous and dense films. Figure 6b shows the chemical elemental spectrum of pure TiO_2_ deposited on the FTO glass, as detected using EDS. In the spectrum, three elements were detected in the pure TiO_2_ film, namely, titanium (Ti), oxygen (O), and carbon (C). Ti and O elements showed high percentages at 32.93% and 63.69%, respectively, which proved that the film on the FTO glass was TiO_2_. The C element at 3.38% was probably due to contaminations during the preparation of FESEM sample. No other components were shown in the spectrum, indicating that the thin film was pure TiO_2_. Figure 6c shows the XRD patterns of AgNP, pure anatase TiO_2_, and Ag-TiO_2_ nanopowder at different AgNP sizes. As shown in the figure, four Bragg diffraction peaks at 2θ, namely, 38.18°, 44.40°, 64.82°, and 77.96° that correspond to the (111), (200), (220), and (311) planes of the face centre cubic (fcc) lattice structure of silver (JCPDS no. 04-0783). In addition, no Bragg peaks relating to other material components were observed. This result showed that the synthesised AgNP was highly purified. For TiO_2_, the characteristic peaks can be observed at 2θ of 25.86° (101), 38.03° (004), 48.52° (200), 54.38° (105), 55.54° (211), 63.28° (204), and 75.62° (215) (JCPDS no. 21-1272). The patterns showed no rutile and brookite phases, indicating that the TiO_2_ was an anatase phase lattice. Figure 6c also shows an increase in the peak at 38°, which was observed for Ag-TiO_2_ compared to pure TiO_2_. It shows the existence of AgNP in TiO_2_, at lattice Ag (111) and TiO_2_ (004). In addition, the intensity at lattice (101) peak of Ag-TiO_2_ was increased. The presence of AgNP showed its high crystallinity films compared to anatase TiO_2_.

### 3.2. Optimisation of Photoanode Thickness

**Figure 7:**
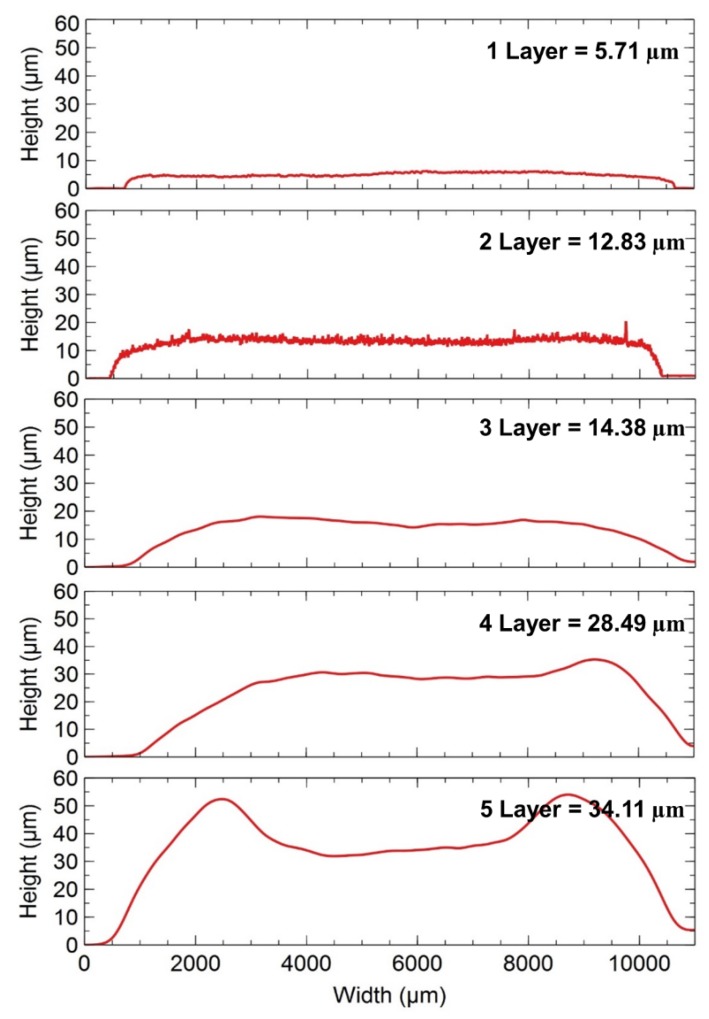
Height profile for different thickness TiO_2_ using doctor blade method. The measured thickness using one layer of tape = 5.71 µm, two layers = 12.83 µm, three layers = 14.38 µm, four layers = 28.49 µm and five layers = 34.11 µm.

**Figure 8:**
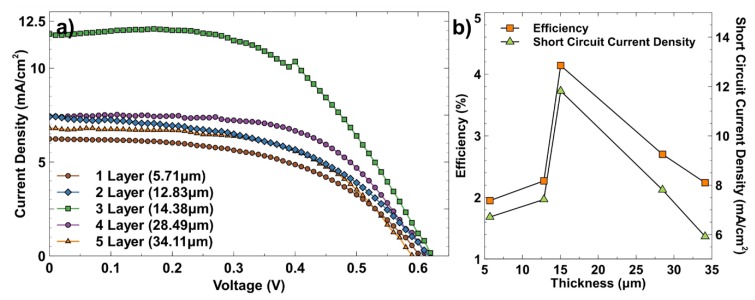
(**a**) Current-Voltage (I–V) characteristic (**b**) power-conversion efficiency and short circuit current density for different TiO_2_ film thickness.

The height profile of TiO_2_ films using different layers of scotch tape, from one layer to five layers, is shown in Figure 7, with a scanning area of 1 cm^2^ using a 3D laser measuring microscope. The measured thickness using one layer of tape = 5.71 µm, two layers = 12.83 µm, three layers = 14.38 µm, four layers = 28.49 µm, and five layers = 34.11 µm, which was the maximum number of layers used in the experiment. As seen in Figure 8, when the thickness was increased, a bowl-like shape became more significant, especially with five layers of Scotch. This result can be attributed to the centre of the film drying faster than the edges during the 125 °C pre-heating on the hotplate, which can reduce the performance of the solar cells due to non-uniform surface.

A comparison of the I–V characteristic of different TiO_2_ film thicknesses is shown in Figure 8a. In DSSC, the thickness of the photoanode is an important parameter to achieve optimal power-conversion efficiency. Thicker films can increase free electron generation and charge recombination. Meanwhile, thinner films can reduce charge recombination and the dye loading on TiO_2_ [9]. Therefore, optimal thickness is required to overcome these problems. As shown in Figure 8a, 14.38 µm or three layers of Scotch tape was able to achieve high circuit current density. Figure 8b shows the performance efficiency (η) and short circuit current density (J_SC_) at different TiO_2_ film thicknesses. The J_SC_ was increased from 6.71 to 11.82 mA/cm^2^ when the thickness of TiO_2_ film was increased and started to reduce when the thickness was further increased to more than 28.49 µm. The power conversion efficiency had also followed the same graph trend as J_SC_, with the highest efficiency of 4.14% for 14.38 µm thickness. The increasing J_SC_ at TiO_2_ thickness from 5.71 to 14.38 µm can be linked to the increased dye loading onto TiO_2_ nanoparticles. Therefore, the rate of electron injection from excited dyes to the conduction band of TiO_2_ nanoparticles had improved and maximised photon adsorption by dye molecules [10,17]. Even though thicker TiO_2_ films can have higher dye loading and absorb more photons that could increase the J_SC_, it could also increase electron recombination with I_3_^−^ ions and induce the loss of injected electrons [10,18]. On the other hand, the increased thickness to more than 28.49 µm was causing the rise of internal series of electron transport resistance, hopping from one TiO_2_ nanoparticle to the next [18]. This problem can lower the J_SC_ value and reduce the efficiency. Thus, 14.38 µm was considered as the optimal thickness for this experiment.

Table 1 shows a summary of the photovoltaic DSSC parameters with different thicknesses of TiO_2_ layers. The open circuit voltage (V_OC_) was slightly increased from 0.61 to 0.63 V when the thickness was increased up to 14.38 µm, and the V_OC_ started to decrease when the thickness was further increased. The increasing thickness would increase the surface area of TiO_2_ that can improve the number of trapping surface states. Thus, the back transfer of electron to I_3_^−^ ions will be enhanced, resulting in the lowering of V_OC_ [10,19]. Table 1 also shows that the highest fill factor (FF) was found at the thickness of 14.38 µm, with FF value of 0.56. As the TiO_2_ film grew thicker, the FF value began to decrease. The increasing FF values could be due to the higher generation of free electrons in the TiO_2_ film, which was causing an increase of maximum power point (Pmax) [20]. Thus, it was concluded that the optimal thickness was 14.38 µm, or three layers of scotch tape, to achieve the highest photocurrent generation and power conversion efficiency. 

### 3.3. Effect of Surface Plasmon Resonance (SPR) at Different AgNP Sizes on the Performance of Dye-Sensitized Solar Cells (DSSC)

**Figure 9:**
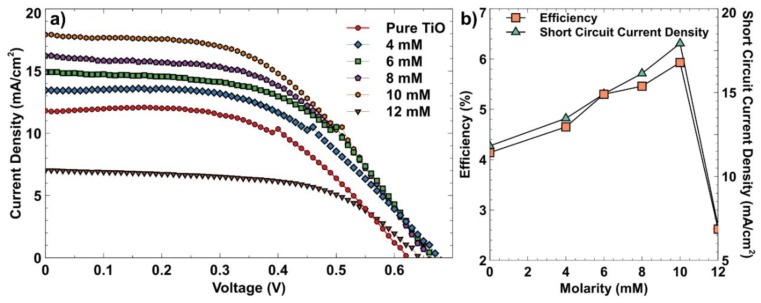
(**a**) I–V characteristic (**b**) power conversion efficiency and short circuit current density for different concentration of sodium citrate.

The effect of SPR on the performance of Ag-TiO_2_-based DSSC was investigated by assembling the prepared photoanodes and measuring I–V characteristic to compare their performance to the pure TiO_2_ photoanode. Figure 9a presents the comparison of I–V curve performances at 1.5 AM irradiation of 100 mW/cm^2^ in between pure TiO_2_ and different AgNP sizes. This figure shows that the 10 mM sodium citrate or the average AgNP size of 65.23 nm was able to achieve a higher J_SC_ compared to pure TiO_2_ and other AgNP sizes. Figure 9b shows the variation of efficiencies and J_SC_ values at different AgNP sizes. The efficiency was increased from 4.14% to 5.93% when the concentration of sodium citrate was increased from 0 to 10 mM and it significantly dropped to 2.62% when the concentration was at 12 mM. The J_SC_ graph similarly followed the same trend as the efficiency, with the highest J_SC_ value of 17.93 mA/cm^2^. Table 2 lists the photovoltaic parameter for different concentrations of sodium citrate. This table shows that with the addition of AgNP onto the TiO_2_, the V_OC_ is improved from 0.63 V for pure TiO_2_ to 0.66 V for 10 mM reducing agent. These results proved that AgNP can enhance light absorption and the generation of charge carriers. The variation of FF values could be due to the discrepancy in electrolyte container thickness. During the assembly of the DSSC device, the TiO_2_ photoanode and counter electrode were sealed together using a 60 µm spacer. Since this process was done manually and to make sure that both electrodes were tightly sealed, there could have been uneven electrolyte container thickness, which resulted in the fluctuating FF values. This method could be improved by using a heat press machine [20] since it offers uniform pressure during the heat press process.

Thus, the main factors that have contributed to the increasing J_SC_ values could be attributed to the enhancement of light absorption. Meanwhile, the broadened light spectrum of the dye (refer to Figure 4) had arisen from the SPR of AgNP, which had enhanced charge carrier generation, electron injection, and electron transport to the TiO_2_ layer [21,22]. However, these enhancements were only limited to AgNP with small particles. A further increase in particle size had only reduced the amount of dye molecules getting adsorbed onto the TiO_2_ nanoparticles, which reduced the photon absorption for electron generation [23,24]. Thus, optimal sizes of AgNP are required to avoid the reduction of dye molecule adsorption. In addition, the Schottky barrier at the Ag-TiO_2_ interface will create electron-hole pairs that would improve the flow of photo-generated electrons through the TiO_2_ network [21]. The Schottky barrier was formed at the Ag-TiO_2_ interface because of the lower work function value of TiO_2_ (semiconductor) compared to the work function of Ag (metal) [25]. When light was irradiated onto the Ag-TiO_2_ film, the electrons from the dye were injected into the CB of TiO_2_. The SPR effect from Ag was responsible for the generation of free electrons, causing an accumulation of electrons. Therefore, some of the light would be absorbed by Ag and electrons with energy higher than the Schottky barrier would cross the barrier and be injected into the conduction band (CB) of TiO_2_. Thus, fractions of electrons from the dye can be directly injected into the CB of TiO_2_ or to the CB through AgNP [26,27]. With the presence of the Schottky barrier, electrons at the CB of TiO_2_ were prevented from going back to the dye molecules or to the electrolyte, which could reduce the recombination rate and improve the J_SC_. In the DSSC, V_OC_ depends on the difference between the Fermi level of Ag and the redox potential of the electrolyte. Therefore, the observed V_OC_ had increased from 0.63 V for pure TiO_2_ to 0.68 V for 46.89 nm AgNP. This result indicated that the quasi-fermi level had shifted to negative, causing an increased V_OC_ [28]. However, when the mean diameter of the AgNP was increased, the V_OC_ began to fluctuate due to the AgNP being gradually corroded by the redox mediator that can increase the recombination process. The recombination process can lead to shorter electron lifetime and lowering of the V_OC_. Therefore, this problem can be mitigated by applying a coating or an insulating layer on AgNP [27].

### 3.4. Reproducibility of DSSC

**Figure 10:**
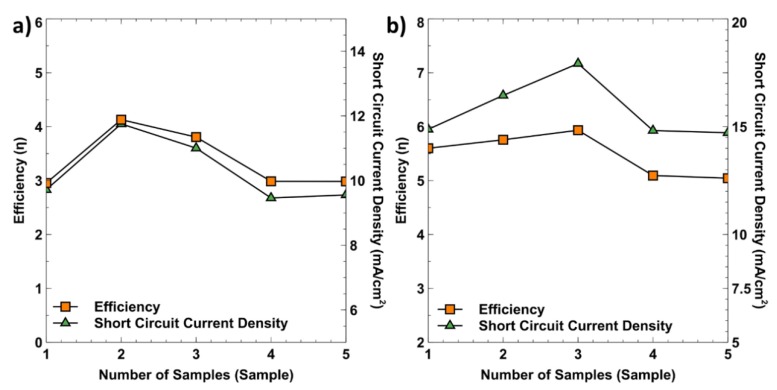
Power-conversion efficiency and short circuit current density for five different samples (**a**) 14.38 nm thickness of TiO_2_ film and (**b**) 65.23 nm mean diameter AgNP to test the reproducibility of the DSSC devices.

To determine the reproducibility of DSSC devices, five different samples were built and tested using the optimised TiO_2_ film thickness and 65.23 nm AgNP. The trend of the power conversion efficiency and the J_SC_ of TiO_2_ thickness and AgNP obtained from the tested devices are shown in Figure 10a,b. Figure 10a shows that all five samples are fabricated using three layers of Scotch tape and deposited onto the FTO glass substrates using the doctor blade method. The minimum efficiency achieved was at 3.0%, with J_SC_ at 9.27 mA/cm^2^, while the maximum efficiency was at 4.14%, as previously mentioned in Section 3.2. The average efficiency of all five samples was at 3.37%. On the other hand, Figure 10b shows the sample with the mean diameter of AgNP of 65.23 nm and three layers of Scotch tape. As observed, the Jsc values were in the range of 14.78% to 17.93%. The average efficiency of 5.64% among these samples showed that the AgNP can help to enhance photocurrent generation. This repoducibility testing has shown that the fabricated DSSC device can be practically implemented in PV technology.

## 4. Conclusions

As a conclusion, the Ag-TiO_2_ nanocomposites were successfully prepared to study the effect of SPR on the performance of DSSC via characterization and I–V characteristic approach. The AgNP was prepared using a simple chemical reduction method and the concentration of sodium citrate was varied to investigate the effect of different nanoparticle sizes. The results showed that as the concentration increases, the mean diameter of the nanoparticles increased as well due to agglomeration. For Ag-TiO_2_ nanocomposites, the highest efficiency was achieved at 6.92% for 10 mM sodium citrate, with a mean diameter of 65.23 nm. The thickness of TiO_2_ had also played an important role to improve the efficiency of the DSSC. The optimal thickness was at three layers of Scotch tape or 14.38 µm, with an efficiency of 4.14%, which was enough to improve the dye loading and reduce charge recombination. Thus, these results can provide invaluable guidance for higher DSSC efficiency. However, this experimental work had only focused on nanoparticle structures. Since SPR is dependent on the size, shape, and dielectric of the plasmonic nanomaterial, further investigation is required based on these properties to provide a deeper and wider understanding on the effect of SPR in DSSC.

## Figures and Tables

**Table 1:** Photovoltaic parameter of dye-sensitized solar cells (DSSC) with different TiO_2_ film thickness including average data with standard deviation were based on five devices. The best performing devices data are given in parentheses.

Scotch Tape (Layer)	Thickness (µm)	V_OC_ (V)	J_SC_ (mA/cm^2^)	FF	η (%)
1	5.71	0.58 ± 0.02 (0.61)	5.74 ± 0.78 (6.71)	0.46 ± 0.03 (0.48)	1.59 ± 0.28 (1.95)
2	12.83	0.62 ± 0.01 (0.62)	6.60 ± 0.70 (7.42)	0.50 ± 0.01 (0.48)	2.07 ± 0.17 (2.27)
3	14.38	0.63 ± 0.02 (0.63)	10.30 ± 1.02 (11.82)	0.52 ± 0.05 (0.56)	3.37 ± 0.56 (4.14)
4	28.49	0.60 ± 0.02 (0.62)	7.83 ± 0.09 (7.81)	0.52 ± 0.02 (0.55)	2.52 ± 0.12 (2.70)
5	34.11	0.60 ± 0.02 (0.62)	6.20 ± 0.40 (5.92)	0.53 ± 0.53 (0.53)	2.16 ± 0.11 (2.24)

**Table 2:** Photovoltaics parameter of DSSC for different sodium citrate concentration.

Reducing Agent Molarity (mM)	Average Diameter (Δnm)	V_OC_ (V)	J_SC_ (mA/cm^2^)	FF	η (%)
0	0	0.63 ± 0.02 (0.63)	10.30 ± 1.02 (11.82)	0.52 ± 0.05 (0.56)	3.37 ± 0.56 (4.14)
4	46.89	0.68 ± 0.01 (0.68)	13.42 ± 0.38 (13.46)	0.51 ± 0.01 (0.51)	4.58 ± 0.11 (4.65)
6	52.39	0.67 ± 0.01 (0.67)	14.62 ± 0.38 (14.92)	0.52 ± 0.01 (0.53)	5.19 ± 0.10 (5.30)
8	58.61	0.66 ± 0.01 (0.66)	15.81 ± 0.35 (16.14)	0.51 ± 0.01 (0.51)	5.35 ± 0.16 (5.46)
10	65.23	0.67 ± 0.01 (0.66)	15.76 ± 1.41 (17.93)	0.51 ± 0.01 (0.51)	5.49 ± 0.40 (5.93)
12	75.54	0.64 ± 0.01 (0.64)	6.73 ± 0.60 (7.03)	0.57 ± 0.00 (0.57)	2.49 ± 0.25 (2.62)
